# Comparison of Fine Needle Aspiration Cytology and Thyroid Scan in Solitary Thyroid Nodule

**DOI:** 10.4061/2011/754041

**Published:** 2011-05-04

**Authors:** Rabia Basharat, Mulazim Hussain Bukhari, Shahzad Saeed, Tahira Hamid

**Affiliations:** ^1^Department of Pathology, King Edward Medical University, Lahore 54000, Pakistan; ^2^King Edward Medical University, Lahore 54000, Pakistan; ^3^Department of Radiology, King Edward Medical University, Lahore 54000, Pakistan; ^4^Fatima Jinnah Medical College, Lahore, Pakistan

## Abstract

*Objective*. This was a comparative study between FNAC and thyroid scan used to diagnose the solitary thyroid nodule and histopathology was used as gold standard to compare the results of both modalities. We hypothesized that Fine needle aspiration cytology and thyroid scan diagnose solitary thyroid nodule (STN) as accurately as histopathology. *Materials and Methods*. This study comprised of 50 patients with solitary thyroid nodules (STN) presented to OPD. After clinical examination these patients were referred to Centre for Nuclear Medicine, Mayo Hospital Lahore for thyroid function tests and thyroid scan (TS). These patients underwent FNAC in the department of Pathology and surgery in Mayo Hospital. The cases were operated and evaluated for histopathological changes. *Results*. On thyroid scan, 40 patients (80%) having cold nodule were labeled as suspicious 10 patients (20%) had hot nodule. On FNAC 23 patients (46%) had benign lesion, 22 patients (44%) had indeterminate lesion and 5 patients (10%) had malignant lesions. On histopathology, 45 patients (90%) were confirmed to have benign lesions and 5 patients (10%), malignant lesions. After comparison of results of thyroid scan and FNAC with histopathology, the sensitivity, specificity, positive predictive value, negative predictive value and diagnostic accuracy of thyroid scan were 80%, 20%, 10%, 90% and 26%, respectively whereas those of FNAC were 80%, 97.7%, 80%, 97.7% and 96%, respectively. *Conclusion*. Fine needle aspiration was a significantly better predictor of malignancy than thyroid scan and resulted in a smaller proportion of excisions for benign nodules.

## 1. Introduction

Thyroid nodular (TN) lesions are a common clinical problem in the world. These are more common in women and in areas of iodine deficiency. Exposure to ionizing radiation in childhood and adolescence increases the risk of solitary thyroid nodule and thyroid carcinoma. In the United States, 4 to 7% of the adult population has a palpable thyroid nodule [1].

A solitary thyroid nodule is a palpable swelling in thyroid gland that has otherwise a normal appearance [2]. The majority of thyroid nodules are asymptomatic and only about 5 percent of all palpable nodules are found to be malignant. A variety of tests have been employed to separate benign from malignant thyroid nodules [2, 3].

These tests include isotope scanning and fine needle aspiration cytology. Combined use of isotope scanning, fine needle aspiration cytology, and histopathology of thyroid offers the best diagnostic strategy [4].

Isotope scanning was generally used to classify nodules into nonfunctioning (cold) or functioning (warm or hot) nodules. The scans used either Iodine123 or technetium Tc99m pertechnetate. Only 5 to 15% of the cold nodules are malignant [1, 5].

As the cost of 131 scintigraphy increases, the I-131 scintigraphy becomes less effective than the fine needle aspiration cytology strategy. In one study of solitary thyroid nodule, the specificity of scan turns out to be only 21.1%. So advice is there to avoid this technique as a routine test in patients with nontoxic thyroid nodules [6, 7] (6, 8).

Fine needle aspiration cytology of thyroid nodules is the single most sensitive, specific, and cost-effective method of investigation of thyroid nodules. Now it is safely and widely recommended for the preoperative selection of patients. The major pitfall of this procedure is that fine needle aspiration cytology cannot differentiate between follicular adenoma and follicular carcinoma [5, 7]. 

Histopathology of the excised specimen showed multinodular goiter as the commonest lesion. In one study, fine needle aspiration cytology and thyroid scan offers the best preoperative assessment of solitary thyroid nodule. Histopathology later on confirms the preoperative diagnosis. As the incidence of solitary thyroid nodule is high in Pakistan, this study will help in early detection of thyroid lesions [8].

Many modalities are used for the diagnosis of solitary nodule in Pakistan but histopathology remained the gold standard for the comparison of all. In a recent study it has been seen that FNAC is a primary investigation of thyroid lesions and used in a patient with one or more thyroid nodule. FNAC is also advised for every patient for exclusion of cancer and in the initial management of patients. It is frequently used because it is inexpensive, sensitive, specific, and an accurate procedure; therefore it is adapted as an initial investigation of thyroid diseases in all tertiary hospitals in developing countries like Pakistan [9]. In Pakistan, before performing manual or guided FNAC, thyroid scan and thyroid function tests (TFTs) are performed for further management of solitary thyroid nodules. In this study we compared the individual efficacy of FNAC and thyroid scan in the management of solitary thyroid nodules. The nodules were marked after doing their scan by the nuclear center of Mayo Hospital taking histopathology as gold standard.

## 2. Material and Methods

This was a comparative cross-sectional study and carried out at the Departments of Surgery, Nuclear Medicine, and Pathology, King Edward Medical University, Lahore. It was conducted on 50 patients of solitary thyroid nodule in 06 months. A nonprobability purposive sampling technique was used for these patients.

**Inclusion Criteria**

1. Age 10 to 70 Years.
2. Both genders.
3. Patient presenting with solitary swelling arising from any lobe of thyroid selected by clinical palpation.

**Exclusion Criteria**

1. Patients with diffuse thyroid swelling.
2. All toxic and multinodular goiters confirmed by clinical evaluation.
3. Patients with history of any type of thyroid surgery (lobectomy or total thyroidectomy).

### 2.1. Data Collection

All patients presenting with solitary thyroid nodules in the OPD and fulfilling the inclusion criteria were included in this study. Informed consent from all the patients included in the study was taken. All the patients were recorded for their demographic features, that is, age, sex, and address (for followup). History of present illness with regard to symptoms and duration was recorded. They were examined for the signs related to the solitary thyroid swelling. All routine investigations and serum T3, T4, and TSH levels were performed by Radioimmunoassay (RIA), (normal range of T3, 2.5–5.8 pmol/L, T4, 11.5–23.0 pmol/L, and TSH, 0.2–4.0 mIU/L). Patients with solitary thyroid swelling underwent thyroid scan. Solitary thyroid nodules were selected and then FNAC was performed. The radionuclide scan and FNA were performed in conjunction as compared to FNAC alone.

Cytological diagnosis was categorized into four groups: negative for malignancy, indeterminate (suspicious) for malignancy, positive for malignancy, and inadequate. The cases were operated and evaluated for histopathological changes. The results of thyroid scan, fine needle aspiration cytology, and histopathology were compared. Histopathology was taken as gold standard. All these information were collected through proforma that is attached herewith.

### 2.2. Statistical Analysis

All the data was analyzed with SPSS version 11. The variables included were demographic information, routine investigations, thyroid scan, and thyroid function tests. For quantitative data, that is, thyroid function tests, duration and size of thyroid nodule, mean, and standard deviation were calculated. For qualitative data, that is, results of thyroid scan, fine needle aspiration cytology, and histopathology, percentages was calculated. A 2 × 2 table was used to calculate sensitivity, specificity, positive predictive value, negative predictive value, and accuracy.

## 3. Results

The age of patients ranged from 10 to 70 years with mean age 33.04 ± 12.29 years. 41 patients (82%) were females, and 9 (18%) were males (male to female ratio 1 : 4.6) (Table 1). 

Regarding thyroid function tests, 48 patients (96%) were euthyroid, 2 patients (4%) were hyperthyroid, and no patient was hypothyroid. The mean for serum T3, serum T4, and serum TSH were 4.00 ± 0.90, 16.47 ± 3.18, and 0.95 ± 0.85, respectively, (normal values given in Table 2). There were 40 patients (80%) who had cold nodule on thyroid scan of which 8 patients (16%) were male and 32 patients (64%) were female, while 10 patients (20%) had hot nodule on thyroid scan of which 1 patient (2%) was male and 9 patients (18%) were female (Tables 2 and 3). 

The mean for size of thyroid nodule was 4.60 ± 1.63 cm. 35 patients (70%) had 1–5 cm sized thyroid nodules, and 15 patients (30%) had 6–10 cm thyroid nodules. No patient had thyroid nodule greater than 10 cm in size (Table 4).

Out of 50 patients, 23 patients (46%) were of benign FNAC (7 colloid cyst colloid, 2 multinodular goiter, 12 colloid goiter, and 2 chronic lymphocytic thyroiditis). 22 patients (44%) were of indeterminant including suspicious for malignancy FNAC of which 2 patients (4%) were males and 20 patients (40%) were females, and 5 patients (10%) were of malignant FNAC of which 1 patient (2%) was male and 4 patients (8%) were female. Out of the 23 patients with benign FNAC, 7 patients (30.4%) had colloid cyst, 2 (8.7%) males and 5 (21.73%) females; 2 patients (8.7%) had colloid goiter, 1 (4.35%) male and 1 (4.35%) female; 12 patients (52.17%) had colloid nodule, 3 (13.04%) males and 9 (39.13%) females, and 2 patients (8.7%) had lymphocytic thyroiditis, both females (Tables 4-5).

Out of the 5 patients with malignant FNAC, 3 patients (60%) had papillary carcinoma, 1 (20%) male and 2 (40%) females; 1 patient (20%) had medullary carcinoma who was female, and 1 patient (20%) had anaplastic carcinoma who was female (Table 4).

Out of the 22 patients with indeterminant FNAC, 3 patients (13.63%) had follicular lesion, all of them females, and 19 patients (86.37%) had follicular neoplasm, 2 (9.1%) males and 17 (77.27%) females (Table 4). 

On histopathology, 45 patients (90%) were confirmed to have benign lesions and 5 patients (10%) malignant lesions. Out of 45 patients with benign lesions on histopathology, 7 patients (15.5%) had colloid cyst, 2 (4.4%) males and 5 (11.1%) females; 2 patients (4.4%) had colloid goiter, 1 (2.2%) male and 1 (2.2%) female; 12 patients (26.67%) had colloid nodule, 3 (6.67%) males and 9 (20%) females; 2 patients (4.4%) had chronic lymphocytic thyroiditis, both females; 18 patients (40%) had follicular adenoma, 2 (4.4%) males and 16 (35.5%) females; 1 patient (2.25%) had diffuse hyperplasia which was female, and 3 patients (6.67%) had hyperplastic nodule, all of whom were females. Out of 5 patients with malignant lesions on histopathology, 2 patients (40%) had pure papillary carcinoma, 1 (20%) male and 1 (20%) female; 1 patient (20%) had medullary carcinoma who was female, 1 patient (20%) had anaplastic carcinoma who was female, and 1 patient (20%) had angioinvasive follicular carcinoma (Table 4).

**Figure 1:**
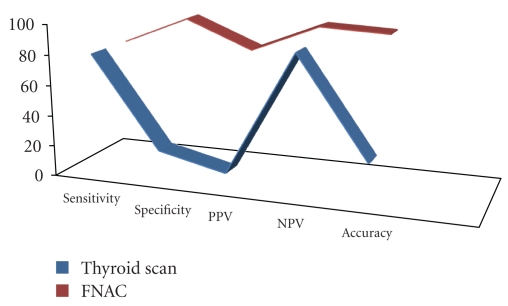
Comparison of sensitivity, specificity, positive predictive value, negative predictive value and diagnostic accuracy of thyroid scan and fine needle aspiration cytology.

On comparison of results of thyroid scan with histopathology taken as gold standard, out of 50 patients, 4 patients were true positive, 9 patients true negative, 36 patients false positive, and one patient false negative (Table 6). The sensitivity of thyroid scan was found to be 80%, specificity 20%, diagnostic accuracy 26%, positive predictive value 10%, and negative predictive value 90% (Figure 1 and Table 8).

On comparison of results of FNAC with histopathology taken as gold standard, out of 50 patients, 4 patients were true positive, 44 patients true negative, one patient false positive, and one patient false negative (Tables 6-7). The sensitivity of FNAC was 80%, specificity 97.7%, diagnostic accuracy 96%, positive predictive value 80%, and negative predictive value 97.7% (Table 8).

**Figure 2:**
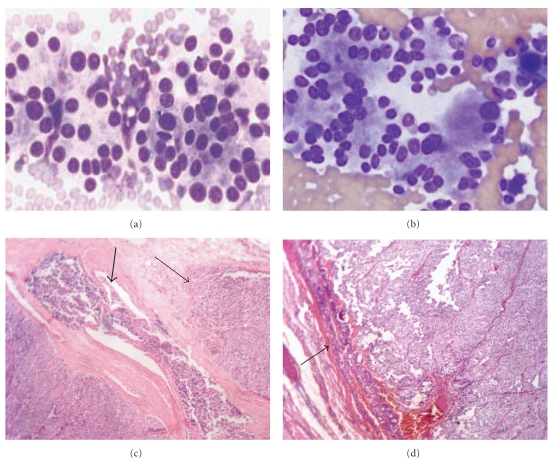
Photomicrograph of FNAC (a) and (b) (H&E) follicular neoplasms, showing marked cellularity, discohesion, single cells, Predominantly microfollicles and/or trabeculae, uniformly enlarged cells, crowding, scant colloid, marked nuclear atypia, mitosis and necrosis is uncommon. (c) Histopathology (H&E 10x) of follicular carcinoma showing capsular (thin arrow) and vascular invasion (thick arrow). (d) Follicular adenoma, (H&E 10x) where no capsular invasion is seen while histologic evidence of invasion is the gold standard of malignancy for the follicular lesions.

**Figure 3:**
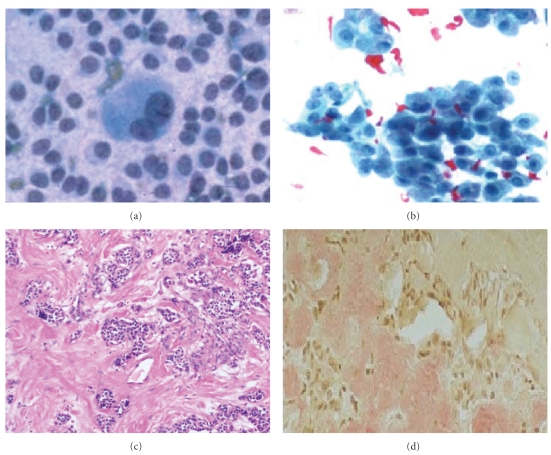
(a) and (b) Photomicrograph on FNAC (Pap) of medullary carcinoma with isolated, loosely cohesive, syncytial fragments, round, oval, cuboidal, plasmacytoid, spindle, round, multinucleated, nuclear inclusions and pale, fibrillar, calcitonin granules and extracellular amyloid. (c) Histopathology (10x H&E) of same case and (d) Congo red positivity.

**Figure 4:**
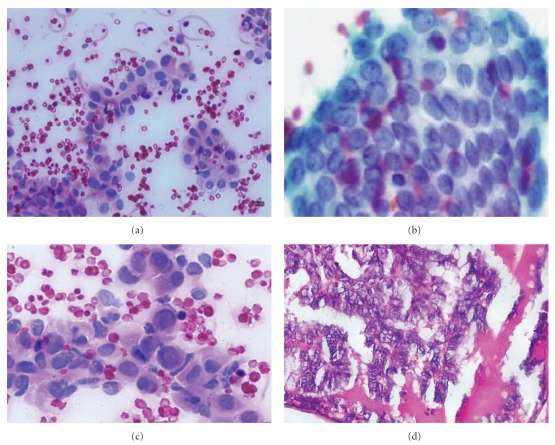
(a), (b), and (c) Photomicrographs of papillary carcinoma showing hypochromasia/pallor, nuclear grooves, intranuclear cytoplasmic inclusions, ovoid nucleus, and micronucleolus. (d) Histopathology of papillary cell carcinoma.

**Figure 5:**
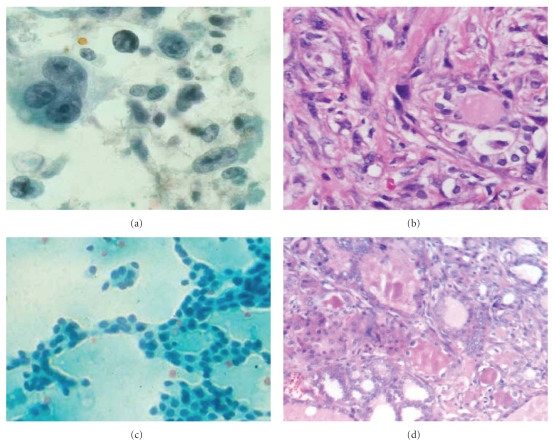
(a) Photomicrograph on FNAC (Pap stain 40x), anaplastic carcinoma showing pleomorphism in size and shape with giant or multinucleate forms, prominent nucleoli, nuclear inclusions, and vacuolated cytoplasm. (b) Histopathology (10x H&E) of anaplastic carcinoma. (c) FNAC (Pap stain 20x) of colloid goiter showing macrophages and uniform cuboidal cells of thyroid follicles. (d) Histopathology of colloid goiter (H&E 40x).

Morphological comparison of different lesions on FNAC and Histopathology is shown in Figures 2–5.

## 4. Discussion

Fine needle aspiration cytology is a well-established technique for preoperative investigation of thyroid nodules. The technique is a noninvasive, cost-effective, and efficient method of differentiating benign and malignant thyroid nodules [1, 10, 11]. Many investigators have shown that fine needle aspiration cytology is the single most sensitive, specific, and cost-effective method in the investigation of solitary thyroid nodules [12, 13]. Despite studies supporting the cost-effectiveness of fine needle aspiration cytology as the diagnostic test of choice, I-131 scintigraphy continues to be used by frontline providers as primary diagnostic tools in the management of patients with nodular thyroid diseases. Justifications for the continued use of this alternative diagnostic strategy usually range from historical practice patterns within institutions to faster turnaround time for results when compared with waiting for FNAC pathology reports. FNAC of thyroid is gaining popularity among pathologists and clinicians all over the world [14–17]. 

In our study, the age of patients ranged from 10 to 70 years with mean age 33.04 ± 12.29 years. The highest number of patients was aged between 21–30 years, that is, 22 (44%). In a study from Saudi Arabia, the mean age was 36.17 ± 12.3 years (range 15–67 years) which is very close to our study [18]. In this study, the female to male ratio was high. In our study, out of 50 patients 41 (82%) were females and only 9 (18%) were males. Female to male ratio was 5.2 : 1. These results are close to Hussain and Anwar, who found female to male ratio as 6.9 : 1 [3]. In our study, 40 patients (80%) had cold nodule on thyroid scan. 10 patients (20%) had hot nodule on thyroid scan. In a study from India, 77.77% patients had cold nodule on thyroid scan while 22.22% patients had hot nodule on thyroid scan, which is close to our study [19].

In the literature, it is clearly indicated that the nodule size is only a weak predictor of histological malignancy [12]. In our study, 35 patients (70%) had 1–5 cm sized thyroid nodules and 15 patients (30%) had 6–10 cm thyroid nodules. The rate of malignancy was almost the same in our data, and the chances of malignancy were independent of its size. Our findings are consistent with other studies [20].

After comparison of our results of thyroid scan with histopathology, overall sensitivity of thyroid scan was found to be 80%, specificity 20%, positive predictive value 10%, and negative predictive value 90%. The overall accuracy was 26%. In one study of thyroid scan, it was reported to have sensitivity 100% and specificity 24% which is close to our study [19]. This shows that thyroid scan is more sensitive than specific in detecting thyroid malignancy. In our study, 23 patients (46%) were of benign FNAC. 22 patients (44%) were of indeterminant FNAC. 5 patients (10%) were of malignant FNAC. In a study from Pakistan, 39.47% of patients were of benign FNAC, 43.42% were of indeterminant FNAC, and 11.84% were of malignant FNAC, which is very close to our study [9].

Out of the 23 patients with benign FNAC, 7 patients (14%) had colloid cyst, 2 patients (4%) had colloid goiter, 12 patients (24%) had colloid nodule, and 2 patients (4%) had lymphocytic thyroiditis. All the patients with benign FNAC were confirmed to have the same results on histopathology. Out of the 22 patients with indeterminant FNAC, 3 patients (6%) had follicular lesion and 19 patients (38%) had follicular neoplasm. On histopathology, all 3 patients with follicular lesions were confirmed to have hyperplastic nodule, and out of the 19 patients with follicular neoplasm, 18 patients (36%) were confirmed to have follicular adenoma whereas 1 patient (2%) had follicular carcinoma. The intermediate findings were the main pitfalls of FNAC thyroid. This could be due to overdiagnosis on cytological reporting [9]. Our findings are consistent with Flanagan et al. [21]. 

Out of the 5 patients with malignant FNAC, 3 patients (6%) had papillary carcinoma (Figure 3), 1 patient (2%) had medullary carcinoma (Figure 2), and 1 patient (2%) had anaplastic carcinoma (Figure 4). On histopathology, out of 3 patients with papillary carcinoma, 2 patients (4%), were confirmed to have papillary carcinoma whereas 1 patient had diffuse hyperplasia. Patients with medullary carcinoma and anaplastic carcinoma on FNAC were found to have the same on histopathology. Therefore, in this study the concordance between the malignant FNAC diagnosis and histologic followup was 80%; this is comparable to other studies in the literature [9, 21–25].

FNAC is a sensitive and highly specific method of evaluating thyroid nodules for malignancy [22–25]. After comparison of our results of FNAC with histopathology, overall sensitivity of FNAC was 80%, specificity 97.7%, positive predictive value 80%, and negative predictive value 97.7%. The overall accuracy was 96%. Our results are consistent with results of other studies. In a review on FNAC of the thyroid nodule, it was reported to have sensitivity of 65–98% and a specificity of 72–100% [26–28]. In another study, the analysis of data revealed a sensitivity of 88.9% and specificity of 96.1% with diagnostic accuracy of 94.2%. This shows that FNAC is more specific than sensitive in detecting thyroid malignancy, and therefore, it is used as a reliable diagnostic test [1].

The false negative rate (FNR) is defined as the percentage of patients with benign cytology in whom malignant lesions are later confirmed on histopathology. Our results about false positive and false negative rates are consistent with the published guidelines of Papanicoloau society of cytopathology [29–31]. This guideline suggested that a false negative and false positive rate of < 2% and 3%, respectively, should be achieved [29]. In series of studies, FNR was reported ranging from 1.5% to 11.5% [32]. Ashcraft and van Herle noted that FNR results varied in reported series from 2% to 50% and that among 1330 patients, all of whom had a histological examination, the FNR was 1.7% [33]. In our series, we reported one case as false negative that translated to 2% FNR. This case was however confirmed on histopathology as follicular carcinoma. Our value is consistent with other studies of Boey and colleagues [34].

The false positive rate (FPR) indicates that a patient with malignant FNAC result was found on histological examination to have benign lesion. Caruso and Mezzaferri [32] reported less than 6% FPR while Campbell and Pillsbury [35] reported 1.2%. In our series, we reported 1 case as malignant on FNAC but it turned out to be diffuse hyperplasia on histopathology. The FPR is 2%, which agreed with other series that range 0–8%.

The overall accuracy for FNAC was 96%, which agrees with other studies of 95% [32]. In our study, sensitivity of FNAC was 80%, which was equivalent to the sensitivity of thyroid scan. However, specificity of FNAC was 97.7% compared to only 20% for thyroid scan. The very low specificity of thyroid scan might make it appear as a superfluous investigation compared to FNAC. The overall accuracy of FNAC was 96% while that of thyroid scan was only 26%.These findings are consistent with the data found worldwide [19]. 

In Pakistan as a routine matter, the evaluation of thyroid is carried out with FNA, the ultrasonography, and scanning with 131I and 99Tc. But before this procedure thyroid scans was the most common test in our setup, used to identify “hot” and “cold” lesions. Hot or warm nodules, about 5%, are seldom malignant, whereas cold or hypofunctional have 10% to 25% chances of being malignant. With the introduction of FNA, people are more relying on this procedure as an elected laboratory test: because it is easy, simple, nontraumatic, and very acceptable to the patients. We cannot undermine the usefulness.

FNAC should be advised for every patient for exclusion of cancer. As FNAC is an inexpensive, sensitive, specific, and accurate procedure, it should be adapted as an initial investigation of thyroid diseases in all tertiary hospitals in developing countries like Pakistan.

## 5. Conclusion

Fine needle aspiration cytology is more specific than sensitive whereas thyroid scan is more sensitive than specific in detecting thyroid malignancy. Fine needle aspiration cytology is highly accurate and better than thyroid scan in the evaluation of solitary thyroid nodule. Therefore, FNAC should be adapted as an initial investigation of thyroid diseases in all tertiary hospitals. The information provided by thyroid scan has no significant bearing in the management of solitary thyroid nodule. FNAC provides useful information and may be used along with other clinical information to decide best form of treatment in a solitary thyroid nodule. The use of FNAC has reduced the number of patients with solitary thyroid nodules undergoing unnecessary surgery and has led to proper planning of surgery in malignant cases.

##  Conflict of Interests

The authors declared that there is no conflict of interests.

## Figures and Tables

**Table 1:** Distribution of subjects by age and sex.

Age	Number	Percentage
Male: Female = 9:41 (18% and 82%)		
10–20	04	8.0
21–30	22	44.0
31–40	14	28.0
41–50	04	8.0
51–60	05	10.0
61–70	01	2.0
Total	50	100.0
Mean ± SD	33.04 ± 12.29	
Male to female ration	1 : 4.6	

**Table 2:** Distribution of subjects by thyroid function tests (RIA method).

	Serum T3		Serum T4		Serum TSH	
	(2.5–5.8 pmol/L)		(11.5–23 pmol/L)		(0.2–4.0 mIU/L)	
	No.	%age	No.	%age	No.	%age
Normal	48	96.0	48	96.0	48	96.0
Increased	02	4.0	02	4.0	0	0
Decreased	0	0	0	0	02	4.0
Total	50	100.0	50	100.0	50	100.0

**Table 3:** Distribution of subjects by thyroid scan.

Thyroid scan	Male		Female		Total	
	No.	%age	No.	%age	No.	%age
Cold nodule	08	16.0	32	64.0	40	80.0
Hot nodule	01	2.0	09	18.0	10	20.0
Total	09	18.0	41	82.0	50	100.0

**Table 4:** Distribution of subjects by size of nodule (*n* = 50).

Size of nodule (cm)	Number	Benign 44	Malignant 6
1–5	35 (70.0%)	32	3
6–10	15 (30.0%)	12	3
>10	0	0	0
Total	50 (100.0%)	44	6
Mean ± SD	4.60 ± 1.63		

**Table 5:** Distribution of subjects by benign and malignant lesions FNAC and histopathology.

Classification (benign = 23 and malignant = 5)	FNAC (*n* = 50)	Histopathology (*n* = 50)
Colloid cyst	07 (30.43%)	7 (14%)
Multi nodular colloid goitre	02 (8.70%)	2 (4%)
Colloid goiter	12 (52.17%)	12 (24%)
Chroinic lymphocytic thyroiditis	02 (8.70%)	2 (4%)
Follicular lesions (neoplasm)	21	Nil
Follicular adenoma	—	18 (36%)
Diffuse hyperplasia	—	1 (2%)
Hyperplastic nodule	—	3 (6%)
Papillary carcinoma	03 (60.0%)	2 (4%)
Medullary carcinoma	01 (20.0%)	1 (2%)
Anaplastic carcinoma	01 (20.0%)	1 (2%)
Folicular carcinoma	—	1 (2%)

**Table 6:** Distribution of subjects by classification of FNAC *n* = 50.

Classification	FNAC			Histopathology
	Male	Female	Total	
	No.	No.		
Benign	06 (12.0%)	17 (34.0%)	23 (46.0%)	44 (88%)
Malignant	01 (2.0%)	04 (8.0%)	05 (10.0 %)	06 (12%)
Indeterminant	02 (4.0%)	20 (40.0%)	22 (44.0%)	—
Total	09 (18.0%)	41(82.0%)	50 (100.0%)	50 (100%)

**Table 7:** Comparison of distribution of subjects by classification of FNAC and histopathology.

Classification	FNAC	Histopathology
Benign	23 (46 %)	44 (88%)
Malignant	05 (10%)	06 (12%)
Indeterminant	22 (44%)	—
Total	50 (100%)	50 (1005)

**Table 8:** Comparison of thyroid scan and FNAC with histopathology.

	Thyroid scan and histopathology		Total	FNAC and histopathology (*n* = 50)		Total
	Positive	Negative		Positive	Negative	
Positive	04 (TP)	36 (FP)	40	04	01	05
Negative	01 (FN)	09 (TN)	10	01	44	45
Total	05	45	50	05	45	50
Sensitivity = 80%, specificity = 20%, accuracy 26%, PPV = 10%, and NPV = 90%				Sensitivity = 80.0%, specificity = 97.7%, accuracy = 96.0%, PPV = 80.0%, and NPV = 97.7%		
